# Dietary sources of animal and plant protein intake among Flemish preschool children and the association with socio-economic and lifestyle-related factors

**DOI:** 10.1186/1475-2891-10-97

**Published:** 2011-09-25

**Authors:** Yi Lin, Selin Bolca, Stefanie Vandevijvere, Herman Van Oyen, John Van Camp, Guy De Backer, Leng H Foo, Stefaan De Henauw, Inge Huybrechts

**Affiliations:** 1 Unit Nutrition and Food Safety, Department of Public Health, Faculty of Medicine and Health Sciences, Ghent University, De Pintelaan 185, B-9000 Ghent, Belgium; 2 Laboratory for Bioinformatics and Computational Genomics (BIOBIX), Faculty of Bioscience Engineering, Ghent University, Coupure Links 653, B-9000 Ghent, Belgium; 3 Scientific Institute of Public Health, Department of Public Health and Surveillance, J. Wytsmanstraat 14, B-1050 Brussels, Belgium; 4 Department of Food Safety and Food Quality, Ghent University, Coupure Links 653, B-9000 Ghent, Belgium; 5 Program of Nutrition, School of Health Sciences, Universiti Sains Malaysia, Health Campus, 16150 Kubang Kerian, Kelantan, Malaysia; 6 University College Ghent, Department of Nutrition and Dietetics, Faculty of Health Care Vesalius, Keramiekstraat 80, B-9000 Ghent, Belgium

**Keywords:** plant protein, animal protein, preschool children, socio-economic status, lifestyle-related factors, Flanders

## Abstract

**Background:**

The aims of this study were to assess the intake of animal, plant and food group-specific protein, and to investigate their associations with socio-economic and lifestyle-related factors in Flemish preschoolers.

**Methods:**

Three-day estimated dietary records were collected from 661 preschoolers aged 2.5-6.5 y (338 boys and 323 girls). Multiple linear regression analysis was used to investigate the association between animal, plant, and food group-specific protein intake and socio-economic and lifestyle factors.

**Results:**

Animal proteins (mean 38 g/d) were the main source of total protein (mean 56 g/d), while mean plant protein intake amounted to 18 g/d. The group of meat, poultry, fish and eggs was the main contributor (51%) to animal protein intake, followed by milk and milk products (35%). Bread and cereals (41%) contributed most to the plant protein intake, followed by low-nutritious, energy-dense foods (21%). With higher educated fathers and mothers as reference, respectively, preschoolers with lower secondary and secondary paternal education had lower animal, dairy-, and meat-derived protein intakes, and those with lower secondary and secondary maternal education consumed less plant, and bread and cereal-derived proteins. Compared to children with high physical activity levels, preschoolers with low and moderate physical activity had lower animal and plant protein intakes. Significantly higher potatoes and grains-, and fish- derived proteins were reported for children of smoking mothers and fathers, respectively, compared to those of non-smoking mothers and fathers.

**Conclusions:**

The total protein intake of Flemish preschoolers was sufficient according to the recommendations of the Belgian Superior Health Council. Parental level of education and smoking status might play a role in the sources of children's dietary proteins.

## Background

Dietary protein is considered an important macronutrient, ideally contributing 10-15% to the total energy supply [1]. Protein is considered the most effective macronutrient in thermogenesis via the regulation of energy intake and satiety [2-6]. Moreover, proteins are vital for human metabolism as a source of essential amino acids [7].

In developing countries, proteins receive special attention due to protein-energy malnutrition among infants and children, which is rare in the Western world [8]. Furthermore, high protein intakes have been associated to chronic diseases such as obesity, metabolic syndrome, hypertension, cardiovascular diseases, type 2 diabetes, and kidney diseases [9-14]. Some studies showed that high consumption of total protein might help reducing body weight and improving bone health [9-11,14], whereas other studies reported a positive association between too high protein intake and children' s BMI-z-score [12].

Due to differences in *e.g.*, amino acid composition between animal and plant proteins, these two protein types act differently and should, therefore, be assessed separately [15,16]. A positive association was found between plant protein intake and health outcomes during childhood [12,15,17,18], whereas inverse associations were observed for animal protein intakes [19,20]. Gunther *et al. *(2007) found that higher animal protein intakes early in life, especially dairy protein intake, may be associated with an unfavorable body composition later in life, resulting in a higher risk for chronic diseases [19]. Recent results from the Dortmund Nutritional and Anthropometric Longitudinally Study suggested that a high animal protein intake during mid-childhood might be associated with an earlier pubertal growth spurt peak height velocity, while a higher plant protein intake, conversely, could delay puberty [21].

Parental involvement plays a critical role in promoting children's health behaviour and dietary habits at an early age [22]. Socio-economic status (SES) such as parental level of education and employment status, and more specifically household income might play a role in children's dietary preferences and choices of food quality [23,24]. Additionally, parental lifestyle might be an important factor in developing children's lifestyle and dietary habits. Recent studies indicated that parental smoking, maternal smoking in particular, is associated with a higher prevalence of overweight and obesity among children and early adolescents [25,26], yet without evidence whether children's dietary habits were influenced or not. Furthermore, studies on the relation between children's level of physical activity and pattern and food sources of protein intakes are currently missing.

Until now, only Guillaume *et al. *(2000) assessed the total protein intake among children aged 6-8 y living in the province of Luxemburg in Belgium [27]. However, no comprehensive data on the contribution from dietary animal and plant protein sources is available among Belgian preschoolers. Moreover, only a few studies report on the relation between dietary animal and plant protein intakes, and SES and lifestyle-related factors. The aims of the present study were, therefore, to evaluate dietary protein intake sources from the animal and plant-based foods in Flemish preschoolers, stratified in age-gender groups, and to identify their most important sources. Moreover, associations of total animal and plant, and food group-specific protein intakes were examined with SES and lifestyle-related factors.

## Methods

### Study population and design

The present study was obtained from the preschool dietary survey in the Flanders region of Belgium (October 2002 - February 2003), in which the usual dietary intake of preschoolers (2.5-6.5 y) was estimated from 3d estimated dietary records (EDR) completed by the parents. The participants were representative of Flemish preschoolers recruited from five provinces, including Antwerp, East-Flanders, West-Flanders, Flemish Brabant, and Limburg [28]. The exclusion criteria were: 1) staying in an institution (*e.g*., a hospital school) that provided the food, 2) not attending school during the whole period of the fieldwork, 3) living abroad (e.g. in the Netherlands) but attending school in Flanders, 4) having no Dutch-speaking parent/proxy, and 5) having an older brother or sister participating in the study. The sampling design and methods have been described in detail previously [28,29], along with the response rate and the representativeness of the study sample (50% response rate and 49% after data-cleaning). In brief, a random cluster sampling design at the level of schools, stratified by province and age, was used. The proportion of the variance explained by schools and classes was low (<3%).

Experienced dietitians performed the fieldwork. The school headmasters, teachers and parents were informed about the study objectives and dietary assessment methods during a school meeting. Oral and written instructions were provided for the recording of foods and drinks consumed by children. Teachers were asked to report what the children consumed at school so that the parents/proxies could include this information in the diaries.

The percentage of underreporters has been described in depth in a previous paper and was shown to be low (<2% of the children when using Goldberg cut-offs adapted for children) [30]. Underreporters were not excluded from the study sample for the present analyses because of low prevalence.

A total of 661 out of 1026 children (64%) who completed 3d EDR, were included in the analysis for the present study. Among the 365 excluded children, 330 children did not complete the 3d EDR correctly and 55 children's gender and/or age were missing.

The Ethical Committee of the Ghent University Hospital granted an ethical approval for the present study. Only children for whom a signed informed consent was obtained from (one of) the parents, were included in the study. More detailed information about the study design can be found elsewhere [29].

### Socio-economic status and lifestyle-related factors

Parents were asked to fill out a questionnaire about the family background such as family size, family situation (two-parent family, one-parent family or special situation), level of parental education (lower secondary education, secondary education or higher education (bachelor, master or above) for mother and father), and employment (both parents employed, one parent employed or both parents unemployed), and lifestyle-related factors such as children's level of physical activity (low, moderate or high), and maternal and paternal smoking (yes/no).

Additionally, parents were asked to provide information on their children's age, gender, dietary habits, general health status, and whether the child had been breastfed or not. Also weight and height of the child were reported by the parents.

### Dietary intake assessment

For the current analyses, only completed data from the 3d non-consecutive EDR were used, excluding diaries containing insufficiently detailed descriptions of food products and/or portion sizes. Only diaries with three completed record days were included (n = 696; 66% of collected diaries). A record day was considered as incomplete if the portion size information was missing for most of the principal meal components (e.g. bread, beverages, etc.) or when the specifications about the food type (e.g. fat or skimmed milk) was missing for most of the principal meal components. Two dieticians with long-standing experience in nutritional epidemiological surveys, carried out this exclusion procedure. The distribution of 3d EDR covered all days of the week and the autumn and winter season.

The food composition data for calculating the estimated protein intakes were derived from the following tables - in order of importance: the Belgian NUBEL [31], the Dutch NEVO [32], and the USDA [33] food composition databases, which used the Kjeldahl method for analysing protein [34].

In total, 936 foods and composite dishes were encoded in the original database. All recipes that were described in depth as individual ingredients in the diaries were encoded as ingredients. However, in order to classify foods easily into the food groups according to the Flemish food-based dietary guidelines (FBDG) [35], eight additional composite dishes had to be disaggregated (fried rice, fried rice with eggs, spaghetti Bolognese, chicken ragout, turkey ragout, lasagna, macaroni with ham and cheese sauce, and stew). Spaghetti Bolognese, for instance, was disaggregated into spaghetti, minced meat, onions, tomatoes, carrots, and margarine, according to the recipe list of the Flemish EPIC-soft version 2004 [36]. However, since the ingredients of pizzas (consumed by sixty-eight children during the three recorded days) and quiches (consumed by two children) were seldom described in the diaries, we decided to categorise these food mixtures as such, instead of disaggregated. After the disaggregating procedures, food items were divided into 57 food subgroups of similar nutrient content or consumption, based on the classification of the Flemish FBDG and the expert opinion of the investigators.

### Statistical analysis

The descriptive analysis of energy and total, animal, and plant protein intakes, corrected for within-person variation, was performed by means of the Multiple Source Method (MSM) [37]. With this method, the total variance was adjusted for the intra-individual variances due to day-to-day variability.

Descriptive statistics of the study population are presented as the mean value or as the frequency distribution and standard deviation (SD) stratified by gender-age. Mean food group-specific energy-adjusted daily intakes were calculated based on the tertiles of total protein intake. The normality and equality of the variances were tested by the Kolmogorov-Smirnov test and Levene's test, respectively. Comparisons of normally distributed data were performed with the Student's *t*-test (between gender and age groups) or ANOVA (between tertiles of intake), whereas the non-parametric Kruskal-Wallis test was used to compare means of non-normally distributed data (between tertiles of intake).

To investigate the associations between total animal and plant, and food group-specific protein intakes, and SES and lifestyle-related factors, multiple linear regression analyses were performed (generalized linear regression (GLM)). Each model included SES (maternal and paternal level of education, parental employment, and family situation) and lifestyle-related factors (preschooler's level of physical activity, and maternal and parental smoking status) and was adjusted for potential confounding factors such as total energy intake, age, gender, nationality, and dietary supplement intake. Two-way interactions between potential confounding factors and independent variables, and between the potential confounding factors were created and examined. In the multiple linear regression analyses (GLM), the categories of higher educated mothers, higher educated fathers, unemployed parents, one-parent families, and non-smoking parents were considered as references. Significance of the associations was evaluated with the Type III Wald Χ^2 ^test. Outliers were removed based on residual plots. The statistical analysis was performed by SPSS software version 15.0 (Statistical Package for Social Sciences for Windows) and statistical significance for all the tests was set at a *P *value of 0.05.

## Results

### Study population

Table 1 describes the socio-economic and lifestyle-related factors, and nutrient intakes of the study population. The majority of the children were living with both parents. Approximately half of the parents had a higher education and about 70% of the children's parents were both employed. 30% of the preschoolers were living in Antwerp, 24% in East-Flanders, 22% in West-Flanders, 15% in Flemish Brabant, and 9% in Limburg (data not shown).

**Table 1:** Socio-economic and lifestyle-related factors, and nutrient intakes of preschoolers in the Flanders preschool dietary survey

Characteristic	Total	Boys (n = 338)	Girls (n = 323)
		n (%)	
Age (n = 661)			
2.5-4 y	197 (29.8)	102 (30.2)	95 (29.4)
4-6.5 y	464 (70.2)	236 (69.8)	228 (70.6)
Socio-economic factors			
*Family situation (n = 659)*			
Two-parents family	632 (95.9)	323 (96.1)	309 (95.7)
One-parent family	23 (3.5)	11 (3.3)	12 (3.7)
Special situation	4 (0.6)	2 (0.6)	2 (0.6)
*Maternal level of education (n = 655)*			
Lower secondary	26 (4.0)	9 (2.7)	17 (5.3)
Secondary	250 (38.2)	127 (38.1)	123 (38.2)
Higher	379 (57.9)	197 (59.2)	182 (56.5)
*Paternal level of education (n = 637)*			
Lower secondary	49 (7.7)	25 (7.7)	24 (7.7)
Secondary	279 (43.8)	146 (45.1)	133 (42.5)
Higher	309 (48.5)	153 (47.2)	156 (49.8)
*Parental employment (n = 634)*			
Both parents employed	439 (69.2)	214 (66.0)	225 (72.6)
One parent employed	157 (24.8)	88 (27.2)	69 (22.3)
Both parents unemployed	38 (6.0)	22 (6.8)	16 (5.2)
Lifestyle			
*Preschooler's level of physical activity (n = 652)*			
Low	246 (37.7)	112 (33.4)	134 (42.3)
Moderate	303 (46.5)	163 (48.7)	140 (44.2)
High	103 (15.8)	60 (17.9)	43 (13.6)
*Parental smoking status*			
Smoking mother (n = 659)	98 (14.9)	54 (8.2)	44 (6.7)
Smoking father (n = 643)	160 (24.9)	82 (12.7)	78 (12.1)

### Total energy, total protein, and animal and plant protein intake

The energy derived from total proteins (mean: 224 kcal/d) contributed for 15.5% to the total energy intake (mean: 1455 kcal/d). Animal protein intake (mean: 38 g/d, range: 10.3-96 g/d) was the main contributor (69%) to total protein intake (mean: 56 g/d, 3.3 g/(kg*d), range: 26-125 g/d), while mean plant protein amounted to 17.5 g/d (range: 7.7-46 g/d). Energy, and total, animal, and plant protein intakes were higher in older preschoolers (4-6.5 y), especially in boys (Table 2). However, when considering the children's body weight, the younger (2.5-4 y) had significantly higher protein intakes than the older (*P *< 0.001). Girls at 4-6.5 y, consumed significantly less energy, and total protein, and animal and plant proteins than their male peers (*P *< 0.001, *P *< 0.001, *P *= 0.013, *P *< 0.001, respectively). Furthermore, in the age group of 2.5-4 y, the animal to plant protein ratio was significantly higher for girls than boys (*P *= 0.025).

**Table 2:** Mean total energy, and total protein, animal and plant protein intakes and their contribution to the energy intakes (n = 661)

**Intake**^**a**^	Total	Boys	Girls	***P***^**b**^
Total energy intake (kcal/d)	Mean ± SD			
2.5-4 y	1408.4 ± 260.4	1441.7 ± 253.0	1372.7 ± 264.9	0.045
4-6.5 y	1474.4 ± 240.0*	1526.3 ± 233.7**	1420.8 ± 235.1	<0.001
Total protein (g/d)				
2.5-4 y	54.8 ± 11.8	55.2 ± 10.5	54.3 ± 13.0	0.405
4-6.5 y	56.4 ± 10.7	58.1 ± 10.3**	54.6 ± 10.9	<0.001
Total protein (g/(kg.d))				
2.5-4 y	3.7 ± 0.9	3.7 ± 0.8	3.7 ± 0.9	0.400
4-6.5 y	3.1 ± 0.8*	3.2 ± 0.7*	3.0 ± 0.8*	0.002
Animal protein (g/d)				
2.5-4 y	37.7 ± 10.9	37.2 ± 9.9	38.3 ± 11.8	0.576
4-6.5 y	38.8 ± 9.7	39.9 ± 9.6**	37.7 ± 9.8	0.013
Plant protein (g/d)				
2.5-4 y	17.0 ± 4.8	18.0 ± 5.6	15.9 ± 3.6	0.002
4-6.5 y	17.6 ± 4.4**	18.3 ± 4.5	17.0 ± 4.2**	0.001
Animal/plant protein ratio				
2.5-4 y	2.4 ± 0.8	2.2 ± 0.9	2.5 ± 0.9	0.025
4-6.5 y	2.3 ± 0.8	2.3 ± 0.7	2.3 ± 0.8	0.533
	Energy % ± SD			
Total protein				
2.5-4 y	15.7 ± 2.1	15.5 ± 2.1	15.9 ± 2.0	0.235
4-6.5 y	15.5 ± 2.1	15.4 ± 2.1	15.5 ± 2.0	0.642
Animal protein				
2.5-4 y	10.8 ± 2.4	10.4 ± 2.3	11.1 ± 2.4	0.043
4-6.5 y	10.6 ± 2.3	10.6 ± 2.3	10.7 ± 2.3	0.699
Plant protein				
2.5-4 y	4.9 ± 1.2	5.1 ± 1.4	4.7 ± 0.9	0.050
4-6.5 y	4.8 ± 0.9	4.8 ± 1.0	4.8 ± 0.8	0.971

SD, standard deviation.

^a^Mean total, animal, and plant protein intakes were adjusted for within-person variability using the Multiple Source Method.

^b^*P *value for mean differences between boys and girls (Student's *t*-test after log transformation)

*Mean value was significantly different from 2.5-4 y old, *P *≤ 0.001 (Student's *t*-test after log transformation).

**Mean value was significantly different from 2.5-4 y old, *P *< 0.05 (Student's t-test after log transformation).

### Food (sub)groups contributing to total, animal, and plant protein intake

The main contributor to the total protein intake among preschoolers was the group of meat, poultry, fish, and eggs, followed by milk and milk products, and bread and cereals (Table 3). The subgroups of meat, game and meat products, milk, flavoured milk drinks, chicken and turkey, and cold cuts from meat products were the five most important contributors to animal protein intake. Bread and cereals contributed most to the plant protein intake, followed by low-nutritious, energy-dense foods, and potatoes and grains. Most of contribution to plant protein intake were derived from the subgroups of bread, rolls, crackers and rice cakes, sweet snacks, potatoes, cooked vegetables, and sugared bread. Noteworthy, the contribution of the group of soy products and soymilk to plant protein was three times higher in boys than girls.

**Table 3:** Contribution from all food groups and subgroups to total, animal, and plant protein intakes (n = 661)

	Total protein				Animal protein				Plant protein			
**Food group and subgroup**^**a**^	Total		Boys	Girls	Total		Boys	Girls	Total		Boys	Girls
	%	**order**^**b**^	%	%	%	**order**^**b**^	%	%	%	**order**^**b**^	%	%
**Beverages (including juices)**	**1.98**		**1.98**	**1.94**	**0.28**		**0.28**	**0.28**	**5.80**		**5.67**	**5.83**
Water	0.00		0.00	0.00	0.00		0.00	0.00	0.00		0.00	0.00
Light beverages	0.03		0.04	0.03	0.00		0.00	0.00	0.10		0.12	0.09
Tea and coffee without sugar	0.00		0.00	0.00	0.00		0.00	0.00	0.00		0.00	0.00
Tea and coffee with sugar	0.01		0.01	0.00	0.01		0.01	0.01	0.00		0.00	0.00
Fruit juice	1.20		1.21	1.23	0.00		0.00	0.00	3.90	6	3.81	4.06
Vegetable juice	0.00		0.00	0.00	0.01		0.00	0.00	0.00		0.00	0.01
Soup, bouillon	0.74		0.73	0.71	0.26		0.27	0.27	1.80		1.74	1.75
Soft drinks	0.00		0.00	0.00	0.00		0.00	0.00	0.00		0.00	0.01
**Bread and cereals**	**12.85**		**13.05**	**12.70**	**0.01**		**0.01**	**0.01**	**41.10**		**41.17**	**42.00**
Bread, rolls, crackers, rice cakes	10.34	4	10.54	10.20	0.00		0.00	0.00	33.00	1	33.26	33.74
Sugared bread	1.39		1.31	1.54	0.01		0.01	0.01	4.50	5	4.12	5.07
Breakfast cereals (ready-to-eat, hot)	1.12		1.20	0.96	0.00		0.00	0.00	3.60	8	3.79	3.19
**Potatoes and grains**	**3.94**		**3.86**	**3.98**	**0.15**		**0.13**	**0.15**	**12.40**		**11.91**	**12.81**
Pasta, noodles	0.94		0.85	1.03	0.00		0.00	0.00	3.00	10	2.68	3.41
Rice	0.36		0.35	0.36	0.00		0.00	0.00	1.20		1.11	1.18
Potatoes	2.64	10	2.66	2.59	0.15		0.13	0.15	8.20	3	8.12	8.22
**Vegetables**	**2.35**		**2.26**	**2.27**	**0.00**		**0.00**	**0.22**	**7.53**		**7.10**	**7.47**
Cooked vegetables	1.82		1.85	1.75	0.00		0.00	0.00	5.80	4	5.82	5.77
Raw vegetables	0.20		0.19	0.20	0.00		0.00	0.00	0.63		0.59	0.65
Vegetarian products (*e.g*. tofu, tempe,...)^c^	0.33		0.22	0.32	0.00		0.00	0.22	1.10		0.69	1.05
**Fruits (sweetened and unsweetened)**	**1.43**		**1.41**	**1.42**	**0.00**		**0.00**	**0.00**	**4.59**		**4.45**	**4.70**
Fresh fruit	1.21		1.18	1.21	0.00		0.00	0.00	3.90	6	3.73	4.01
Canned fruit	0.09		0.10	0.09	0.00		0.00	0.00	0.30		0.31	0.30
Dried fruit	0.02		0.01	0.02	0.00		0.00	0.00	0.05		0.04	0.06
Olives	0.00		0.00	0.00	0.00		0.00	0.00	0.00		0.00	0.01
Nuts and seeds	0.11		0.12	0.10	0.00		0.00	0.00	0.34		0.37	0.32
**Soy products and soymilk**	**1.13**		**1.70**	**0.53**	**0.02**		**0.01**	**0.03**	**3.63**		**5.38**	**1.66**
Soy drinks	0.99		1.53	0.44	0.00		0.00	0.00	3.20	9	4.84	1.44
Soy-based desserts	0.13		0.17	0.07	0.00		0.00	0.00	0.43		0.54	0.22
Fermented milk or soy drinks (*e.g. *actimel, yakult,...)	**0.01**		**0.00**	**0.02**	**0.02**		**0.001**	**0.03**	**0.00**		**0.00**	**0.00**
**Milk and milk products**	**24.53**		**24.24**	**24.84**	**35.14**		**34.94**	**35.00**	**1.87**		**1.75**	**1.91**
Milk (including goat's milk)	11.31	2	11.20	11.44	16.60	2	16.57	16.53	0.00		0.00	0.00
Flavoured milk drinks (*e.g. *fristi, chocolate milk,...)	10.48	3	10.20	10.68	14.60	3	14.36	14.67	1.70		1.57	1.74
Yoghurt	0.35		0.34	0.36	0.52		0.50	0.52	0.00		0.00	0.00
Sugared or aromatised yoghurt	1.01		1.12	0.94	1.50		1.62	1.34	0.07		0.07	0.06
Milk desserts	1.37		1.37	1.40	1.90	9	1.87	1.92	0.10		0.09	0.11
Cream	0.01		0.00	0.02	0.02		0.01	0.03	0.00		0.00	0.00
**Cheese**	**7.46**		**7.11**	**7.78**	**10.87**		**10.44**	**11.15**	**0.11**		**0.11**	**0.11**
Hard cheese (no cream cheese)	5.16	7	4.71	5.50	7.60	6	6.97	7.95	0.00		0.00	0.00
Fresh cheese	1.61		1.70	1.56	2.30	8	2.46	2.21	0.10		0.11	0.10
Cheese spread	0.69		0.70	0.72	0.97		1.01	0.99	0.01		0.00	0.01
**Fat and oil**	**0.08**		**0.08**	**0.07**	**0.03**		**0.03**	**0.02**	**0.20**		**0.20**	**0.20**
Butter, margarine	0.08		0.08	0.07	0.03		0.03	0.02	0.20		0.20	0.20
Oil	0.00		0.00	0.00	0.00		0.00	0.00	0.00		0.00	0.00
Frying oil	0.00		0.00	0.00	0.00		0.00	0.00	0.00		0.00	0.00
**Meat, poultry, fish, and eggs**	**34.66**		**34.78**	**34.84**	**50.58**		**50.74**	**49.95**	**0.39**		**0.35**	**0.43**
Meat, game, meat products	15.58	1	15.75	15.56	23.12	1	23.00	22.34	0.06		0.05	0.08
Chicken, turkey	8.01	5	8.28	7.85	11.50	4	11.97	11.24	0.01		0.01	0.00
Fish, shellfish	2.99	9	3.01	2.87	4.20	7	4.33	4.01	0.30		0.27	0.33
Cold cuts from meat products	6.52	6	6.27	6.84	9.50	5	9.27	9.88	0.02		0.02	0.02
Cold cuts from fish products	0.31		0.27	0.31	0.46		0.40	0.44	0.00		0.00	0.00
Eggs^d^	1.26		1.20	1.41	1.80	10	1.77	2.04	0.00		0.00	0.00
**Restgroup (snacks and desserts)**	**8.93**		**8.89**	**8.88**	**3.01**		**2.94**	**3.07**	**21.43**		**20.95**	**21.81**
Brioches	0.43		0.46	0.36	0.01		0.01	0.02	1.37		1.43	1.15
Sweet snacks (*e.g. *waffle, apple pie)	4.97	8	5.02	4.90	1.30		1.30	1.34	12.50	2	12.28	12.74
Salty snacks (*e.g*, cheese biscuits)	0.24		0.20	0.27	0.04		0.03	0.04	0.67		0.57	0.78
Salty sauces	0.56		0.52	0.58	0.48		0.44	0.52	0.71		0.69	0.71
Sweet sauces	0.00		0.00	0.01	0.00		0.00	0.00	0.01		0.00	0.02
Chocolate	0.43		0.38	0.47	0.23		0.20	0.25	0.84		0.74	0.96
Chocolate spread	0.96		0.95	0.95	0.51		0.50	0.51	1.97		1.94	1.98
Other sweet spread (*e.g*. jam, honey, ...)	0.05		0.04	0.05	0.00		0.00	0.00	0.15		0.14	0.18
Sugar	0.00		0.00	0.00	0.00		0.00	0.00	0.00		0.00	0.00
Fried snacks	0.01		0.01	0.02	0.00		0.00	0.00	0.00		0.00	0.00
French fries, croquettes	0.93		0.92	0.96	0.01		0.00	0.02	3.00		2.91	3.12
Sweet desserts (*e.g*. ice cream, tiramisu, ...)	0.35		0.39	0.31	0.43		0.46	0.37	0.21		0.25	0.17
**Miscellaneous**	**0.64**		**0.60**	**0.71**	**0.36**		**0.30**	**0.40**	**0.96**		**0.94**	**1.02**
Pizza	0.38		0.38	0.36	0.31		0.30	0.30	0.52		0.55	0.51
Others ^e^	0.26		0.22	0.35	0.05		0.00	0.10	0.44		0.39	0.51

^a^These mean food group and subgroup-specific intakes are rough estimates calculated from the raw data on which these nutrient contributions are based. The high number of non-consumers in some of the food (sub)groups hindered the adjustment for within-person variation.

^b^Ranking of the 10 food subgroups with the highest contribution to the total, animal or plant protein intakes.

^c^Includes tofu, quorn and pulses.

^d^Includes eggs reported separately and eggs included in disaggregated food mixtures.

^e^Includes foods or components with negligible contributions to the total nutrient intakes that could not be categorized in the above food (sub)groups (*e.g*. quiches herbs and spices, monosodium glutamate, starch, plain gelatin, artificial sweeteners, pectin and cocoa powder).

The energy-adjusted daily intake of beverages, bread and cereals, and restgroup decreased based on the tertiles of total protein intake for both genders. The intake of dairy products, and meat, poultry, fish and eggs, however, increased significantly for both genders (*P *< 0.001; Table 4).

**Table 4:** Mean energy-adjusted daily intakes (g/d) of different food groups based on tertiles^1^of total protein intake (n = 661)

	Total			Boys			Girls			***P***^**a**^		
Food groups	T1	T2	T3	T1	T2	T3	T1	T2	T3	Total	Boys	Girls
Beverages	279.0	243.9	225.4	271.0	249.9	227.9	284.4	236.6	221.8	0.001	0.116	0.008
Bread and cereals	61.5	61.0	56.4	62.7	62.7	57.9	60.7	58.8	54.2	0.028	0.165	0.087
Potatoes and grains	59.7	60.3	60.0	62.4	58.7	59.3	57.9	62.3	60.9	0.976	0.637	0.529
Vegetables	46.2	45.6	51.2	45.2	42.8	53.6	46.9	48.9	47.7	0.256	0.028	0.435
Fruits	69.2	72.3	82.0	72.6	65.3	79.7	66.9	80.7	85.3	0.037	0.095	0.073
Soy products and soymilk	9.6	13.9	15.0	12.3	18.3	25.0	7.8	8.6	1.1	0.763	0.516	0.253
Dairy products^b^	239.2	295.4	362.2	223.4	285.9	351.2	250.0	306.8	377.5	<0.001	<0.001	<0.001
Meat, poultry, fish and eggs	52.7	60.0	69.7	52.8	58.6	67.9	52.7	61.8	72.3	<0.001	<0.001	<0.001
Restgroup	117.9	98.5	92.4	133.0	105.3	102.0	107.6	90.2	79.0	0.014	0.033	0.045

^1^Tertiles based on total protein intake (g/d) among Flemish preschoolers. Tertile 1 (T1): total protein intake <51 g/d; tertile 2 (T2): 52 g/d < total protein intake <60 g/d; tertile 3 (T3): total protein intake ≥ 60 g/d.

^a^*P *value for mean differences between T1, T2, and T3 (ANOVA for the food groups beverages, bread and cereals, potatoes and grains, dairy products, and meat, poultry, fish and eggs; Kruskal-Wallis test for the food groups vegetables, fruits, soy products and soymilk, and restgroup).

^b^Dairy products include the food groups milk and milk products, and cheese.

### Associations between protein intakes and socio-economic status and lifestyle-related factors

Associations between animal, plant, and food group-specific protein intakes, and socio-economic and lifestyle-related factors were examined after adjustment for potential confounding factors including energy intake, gender, age, nationality, and dietary supplement use, and with higher educated parents, preschoolers with a high level of physical activity, families with both parents unemployed, one-parent families, and non-smoking parents as references (Table 5). Inverse associations were found between animal protein intake and the paternal level of education, and the preschoolers' level of physical activity and between plant protein intake and the maternal level of education, and the children's level of physical activity. Additionally, protein intakes derived from dairy and meat sources were inversely associated with the paternal level of education, whereas, compared to children of higher educated mothers, preschoolers of lower secondary or secondary educated mothers had higher animal protein intakes through the consumption of poultry and fish. Moreover, fish-derived protein intakes were also positively associated with smoking fathers. Bread and cereal-, and vegetable-derived protein intakes were inversely associated with the maternal level of education, whereas potato and grain-derived protein intakes were positively associated with smoking fathers. Vegetable- and fruit-derived protein intakes were also inversely associated with the paternal level of education. Children with one employed parent consumed more vegetable proteins compared to those with both parents being unemployed. Furthermore, the inclusion of interaction terms in the model showed significant interactions, between maternal/paternal level of education * total energy intake and between smoking mother*total energy intake. These interactions show that total energy intake and SES and lifestyle factors affect each other's impact on the outcome variables (animal, plant protein and protein sources (meat, poultry, bread and cereal, and potato and grain)) (data not shown).

**Table 5:** Associations between protein intakes and socio-economic and lifestyle-related factors by multiple linear regression

	Animal protein and specific main sources																			
**Predictor variables**^**a,**^	Total animal protein				**Dairy protein**^**b**^				**Meat protein**^**c**^				Poultry protein				Fish protein			
	β	95% CI		P	β	95% CI		P	β	95% CI		P	β	95% CI		P	β	95% CI		P
Lower secondary educated mother	15.68	-12.20	43.55	0.270	-3.00	-6.39	0.39	0.083	-6.33	-15.37	2.71	0.170	0.30	-9.79	10.40	0.953	1.11	0.16	2.05	0.022
Secondary educated mother	6.42	-4.12	16.96	0.233	-0.13	-1.56	1.30	0.859	0.22	-3.64	4.08	0.910	3.96	0.04	7.89	0.048	0.12	-0.25	0.49	0.534
Lower secondary educated father	-1.22	-4.05	1.62	0.400	-4.14	-6.53	-1.74	0.001	-8.65	-15.58	-1.71	0.015	-3.26	-15.76	9.24	0.609	-1.71	-7.62	4.20	0.570
Secondary educated father	-1.77	-3.40	-0.15	0.032	-1.94	-3.22	-0.66	0.003	-3.77	-7.17	-0.37	0.030	-4.47	-10.83	1.89	0.168	-0.55	-3.55	2.46	0.722
Both employed parents	2.88	-27.05	32.80	0.850	-0.57	-3.49	2.35	0.701	11.27	-1.42	23.97	0.082	0.54	-0.82	1.90	0.438	5.64	-1.47	12.76	0.120
One employed parent	1.33	-30.31	32.96	0.934	0.91	-2.21	4.03	0.567	11.47	-1.95	24.89	0.094	0.07	-1.38	1.52	0.922	7.19	-0.33	14.72	0.061
Two- parent family	4.57	-56.85	65.99	0.884	4.17	-2.04	10.37	0.188	-0.98	-27.04	25.07	0.941	5.95	-24.94	36.84	0.706	5.62	-8.98	20.23	0.451
Light physical activity	-4.42	-7.51	-1.33	0.005	-1.20	-3.04	0.65	0.204	0.06	-0.77	0.89	0.892	2.30	-6.24	10.83	0.598	-1.37	-6.53	3.79	0.602
Moderate physical activity	-3.39	-6.47	-0.31	0.031	-0.94	-2.72	0.85	0.305	-0.08	-0.88	0.72	0.839	-4.07	-12.25	4.11	0.330	-4.41	-9.41	0.60	0.084
Smoking mother	7.44	-10.50	25.38	0.416	-0.83	-2.81	1.16	0.414	1.03	-6.58	8.64	0.791	-2.59	-11.61	6.43	0.574	1.93	-2.34	6.20	0.375
Smoking father	7.49	-5.02	20.00	0.241	-0.22	-1.83	1.39	0.788	-0.47	-5.78	4.84	0.862	-2.56	-8.85	3.73	0.426	0.67	0.11	1.23	0.019
	**Plant protein and specific main sources**																			
	**Total plant protein**				**Cereal protein**				**Potato and grain protein**				**Vegetable Protein**				**Fruit Protein**			
Lower secondary educated mother	-14.29	-23.43	-5.15	0.002	-9.67	-15.19	-4.15	0.001	0.58	-2.86	4.03	0.741	-0.12	-0.40	0.17	0.421	-0.25	-0.65	0.15	0.223
Secondary educated mother	-4.08	-7.63	-0.53	0.024	-0.96	-3.07	1.15	0.373	-0.34	-1.79	1.11	0.647	-0.15	-0.26	-0.03	0.012	-0.03	-0.19	0.14	0.750
Lower secondary educated father	-2.62	-13.97	8.73	0.652	0.47	-0.31	1.26	0.239	-2.30	-5.04	0.44	0.099	-0.14	-0.34	0.06	0.159	-0.16	-0.43	0.11	0.248
Secondary educated father	3.41	-2.36	9.19	0.247	0.21	-0.27	0.69	0.397	0.31	-1.08	1.70	0.663	-0.14	-0.25	-0.02	0.020	-0.22	-0.36	-0.07	0.004
Both employed parents	-0.02	-1.45	1.40	0.975	-2.18	-10.31	5.96	0.600	-1.76	-5.06	1.54	0.295	0.05	-0.17	0.27	0.657	-0.01	-0.34	0.31	0.928
One employed parent	0.26	-1.26	1.78	0.738	0.15	-8.45	8.75	0.973	-1.97	-5.45	1.52	0.269	0.25	0.02	0.49	0.034	0.02	-0.32	0.37	0.900
Two- parent family	-0.91	-2.52	0.69	0.265	-2.12	-18.81	14.57	0.804	-3.11	-9.88	3.65	0.367	-0.16	-0.63	0.30	0.490	-0.01	-0.69	0.67	0.981
Light physical activity	-1.61	-6.17	2.94	0.488	0.96	-2.00	3.92	0.524	-0.10	-0.32	0.11	0.347	-0.07	-0.21	0.08	0.373	-0.09	-0.30	0.12	0.399
Moderate physical activity	-4.93	-9.40	-0.45	0.031	-1.05	-3.94	1.83	0.474	-0.14	-0.35	0.08	0.212	0.03	-0.11	0.17	0.672	0.02	-0.18	0.22	0.830
Smoking mother	0.77	-7.42	8.96	0.854	-0.20	-5.08	4.67	0.936	1.34	0.11	2.63	0.033	0.09	-0.06	0.24	0.232	-0.03	-0.24	0.19	0.811
Smoking father	-1.58	-7.29	4.14	0.588	-0.38	-0.84	0.07	0.098	0.40	-0.98	1.78	0.568	0.03	-0.10	0.15	0.690	-0.08	-0.25	0.09	0.353

β: coefficient β; CI, confidence interval; NS, not statistically significant.

^a^GLM was controlled for total energy intake, age, gender, nationality, and dietary supplement intake. Higher educated parents, families with both parents unemployed, preschoolers with a high level of physical activity, and non-smoking parents were used as references.

^b^Dairy products include the food groups milk and milk products, and cheese.

^c^Meat refers to the food subgroup meat, game, and meat products.

## Discussion

To the best of our knowledge, this is the first study that assessed dietary animal and plant protein intake from different food sources in Flemish preschoolers. Moreover, this is the first study to investigate associations between animal, plant, and food group-specific protein intakes, and SES and lifestyle-related factors. The current study shows that the most important contributor to total protein intake among Flemish preschoolers was meat, followed by dairy products, and bread and cereals.

### Total energy, and animal, plant, and food group-specific protein intake

Our results show that all but one boy (4-6.5y) met the recommended dietary allowances (RDA) for protein intake set by the Belgian Superior Health Council (BSHC) (2.5-4 y: 0.86-0.97 g/(kg*d), 4-6.5 y: 0.85-0.91 g/(kg*d)) [38], and the WHO/FAO/UNU (10.0-15.0% of total energy) [1]. Moreover, 58% preschoolers (2.5-4 y: 118 children; 4-6.5 y: 265 children) exceeded the RDA for total protein intake.

A previous Belgian study, also using 3d EDR among 6-8 y old children living in the province of Luxembourg, reported considerably higher dietary energy and total protein intakes (boy: 2308 kcal/d, girls: 2254 kcal/d; 67 g/d for both genders) than our study results (boys: 1501 kcal/d, girls: 1407 kcal/d; boys: 57 g/d, girls: 55 g/d) [27]. Due to the lack of information on total, animal, and plant protein intakes in Belgian preschoolers, we relate our findings to those available on other countries including UK (4d weighted-food records), Germany (3d EDR), Spain (two 24h recalls), and the US (combination of 24h recall and 2d food records) [19,39-41]. Compared to our study population, lower energy intakes were reported for German children aged 1.5-6 y (928-1398 kcal/d) [19]and British children aged 1.5-4.5 y (boys: 1175 kcal/d; girls: 1098 kcal/d) [39], but in Spain (1595 kcal/d) [40]and the US (boys: 1458-1728 kcal/d; girls: 1356-1576 kcal/d) [41], children aged 2-5 y had higher intakes. When comparing the total protein intakes between these populations, a pattern similar to that of the energy intake was observed for the total protein intakes as such, but not for the energy contribution from total protein. On the one hand, total protein intakes observed in the present study were comparable to those among children in the US (boys: 50-61 g/d; girls: 49-51 g/d) [41], but higher than those reported for British (boys: 36 g/d; girls: 33 g/d) [39]and lower than those of Spanish children (66 g/d) [40]. On the other hand, the energy percentage from total protein contributed more in our study population than for German (12.4-13.8%) [19]and British (boys: 12.1%; girls: 12.0%) [39]children. Furthermore, the relative energy contributions from animal and plant proteins were also lower in German children (7.8-9.3%, 4.2-4.5%, respectively) [19].

Concerning the food sources contributing to total protein intake, energy percentage of protein-derived from meat, dairy (including cheese), and bread and cereals (including bread, breakfast cereals, pasta, rice, and flour) were higher in the present population (3.5%, 4.8%, 2.7%, respectively) (data not shown) compared to the German preschoolers (2.5-2.7%, 3.5-4.1%, and 2.4-2.7%, respectively) [19]. Milk and flavoured milk drinks-, meat-, poultry-, pasta-, and French fries and chips-derived protein intakes of Flemish preschoolers were considerably lower than those of American children (25%, 17.4%, 10.3%, 2.7%, and 0.8%, respectively) [41], but substantially higher than those of Spanish children (10.2%, 10.7%, 4.5%, and 4.2%, respectively) [42]. On the other hand, the contributions from yoghurt, and fish and shellfish to the total protein intake were significantly lower in Belgium than in Spain (6.9% and 4.3%, respectively), but much higher than in the US (1.1% and 1.5%, respectively). Additionally, eggs, breakfast cereals, pasta, and nuts and seeds contributed much more to the total protein intake in the US (2.8%, 3.1%, 2.7%, and 2.5%, respectively) than in Belgium, while cold cuts from meat products contributed much less in the US (1.0%). Conversely, bread and cheese contributed considerably more to the total protein intake in Belgium than in the US (10.0% and 5.8%, respectively) and Spain (6.5% and 4.3%, respectively). Noteworthy, legumes contributed substantially to the total protein intake of Spanish children (3.9%), but it had similar contribution to Belgian (1.1%) and US children (1.0%).

To summarize, we observed that the food sources contributing to protein intake in Flemish preschoolers were mainly from animal origin such as meat, game, meat products, milk and cheese, and low-nutritious, energy-dense food (sweet snack in particular), whereas plant sources, including vegetables, fruit, and breakfast cereals, had much lower contributions. Therefore, according to the literature, Flemish preschoolers do not have very healthy dietary habits compared to other countries, as they consume more unhealthy foods such as French fries and chips, cold cuts from meat products, and less vegetables, fruit, legumes, cereals, and fish. Hence, the food sources contributing to the total protein intake among Flemish preschoolers are narrow and limited.

### Associations between animal, plant, and food group-specific protein intakes and socio-economic status and lifestyle-related factors

To the best of our knowledge, there is no data available on the associations between SES and lifestyle-related factors, and dietary protein intakes derived from animal and plant-based foods in general or from particular food groups among Flemish preschoolers.

In this study, with higher educated fathers and mothers as reference respectively, children with (lower) secondary educated fathers had lower animal, dairy-, meat-, vegetable-, and fruit-derived protein intakes, whereas children with (lower) secondary educated mothers consumed less plant, bread and cereal-, and vegetable-derived proteins and more poultry- and fish-derived proteins. Additionally, paternal and maternal smoking were positively associated with fish-, and potato and grain-derived protein intakes, respectively. These findings are in line with previous studies indicating that parental SES and lifestyle-related factors directly influence children's dietary behavior [23,24,43,44]. Recent studies show that children from parents with a higher SES, are more likely to have healthier protein patterns (more cereals, fruit and vegetables), whereas more unhealthy protein patterns, defined by a high proportion of animal proteins, are found in families with a lower SES [23,45-47]. Furthermore, the choice of good-quality protein sources may be a critical factor as well due to differences in amino acid content. Apparently, due to the higher cost, families with a high SES purchase more high-quality food such as lean meat, fish, vegetables and fruits than families with a low SES [24]. In our study, children with one employed parent consumed more vegetables proteins than those with both parents being unemployed.

Furthermore, we found that children having a low or moderate level of physical activity had lower animal and plant protein intakes than those having high levels of physical activity. Moreira *et al. *(2010) reported that sport activities were positively associated with the dietary intake of fish, meat, eggs, vegetables, bread, yoghurt, and cheese [48]. Children's level of physical activity, however, might be influenced by parental education [48,49]. Aranceta *et al. *(2003) found, after controlling for parental education, that children of less educated mothers spending more than 2 h/d on television watching were more likely to follow the 'Snack' pattern [48]. Therefore, parental level of education, maternal in particular, plays an important role in the development of children's eating behavior and lifestyle [49]. Yannakoulia *et al. *(2008) suggested that children in two-parent families have more chances to have regular meals and healthy foods than children from divorced parents [50].

### Strengths and limitations

The present study was the first investigating dietary animal and plant protein intakes in Flemish preschoolers. The results of this large cross-sectional study represent the Flemish preschoolers' animal and plant protein intakes with a good accuracy and validity because of the low prevalence of underreporters (2%) and the high coverage of all five Flemish provinces. Like all studies, some limitations should also be taken in to consideration.

First, the method of 3d EDR represents only the individual children's short-term daily intake rather than usual intake. However, we corrected for within-person variability by using the MSM method to get more precise usual daily protein intakes. Under- or overestimation might influence the true portion sizes, which makes the estimated animal and plant protein intakes less accurate.

In addition, it should be noted that the food composition data, used for calculating dietary protein intake, might as well have introduced some bias in the estimated nutrient contributions [51].

Furthermore, data of SES and lifestyle-related factors were reported by the preschoolers' parents. Therefore, we had to rely on the parents' memory and ability to estimate some lifestyle-related factors such as the frequency and duration of preschoolers' physical activity. Additionally, parents needed to estimate the level of physical activity based on their own definition. Selection bias might lead to bias of imprecise associations [28]. For example, lower educated parents might be unwilling to report their highest level of education.

## Conclusions and recommendations

To conclude, the total dietary protein intake of almost all preschoolers met the RDA of the Belgian Superior Health Council. Meat was the most important contributor to total protein intake, followed by dairy, and bread and cereals. Furthermore, the results show that animal and plant protein intakes were inversely associated with the paternal and maternal level of education, respectively, and children's level of physical activity, and that some food-group specific protein intakes were associated with the parental level of education, smoking and/or employment status. SES and lifestyle-related factors, parental education in particular, seem, therefore, to play a role in the development of children's dietary animal and plant protein intakes.

Although the total protein intakes reached the RDA of the BSHC and WHO, we noticed that the food sources in our study population were narrow and mainly from animal origin rather than from plants. Hence, parental involvement could help to establish healthy food choices in preschoolers. It is important to inform and educate lower educated parents about healthy food habits for their children since an early health-related knowledge and lifestyle can be adopted under parental influence [52]. However, pressuring children to eat and restricting access to specific foods is not recommended because it often leads to overeating, dislikes, and interest in forbidden items [52].

## Abbreviations

SES: socio-economic status; EDR: estimated dietary records; FBDG: Flemish food-based dietary guidelines; MSM: Multiple Source Method; SD: standard deviation; RDA: recommended dietary allowances; BSHC: Belgian Superior Health Council; CI: confidence interval

## Competing interests

The authors declare that they have no competing interests.

## Authors' contributions

YL and IH designed this study and were responsible for the data analyses and the drafting of the manuscript. IH, GDB and SDH were responsible for the study protocol and fieldwork. All authors contributed to the interpretation of the results. All authors read and approved the final manuscript.
